# Highly Aligned Polymeric Nanowire Etch-Mask Lithography Enabling the Integration of Graphene Nanoribbon Transistors

**DOI:** 10.3390/nano11010033

**Published:** 2020-12-25

**Authors:** Sangheon Jeon, Pyunghwa Han, Jeonghwa Jeong, Wan Sik Hwang, Suck Won Hong

**Affiliations:** 1 Department of Cogno-Mechatronics Engineering, Department of Optics and Mechatronics Engineering, College of Nanoscience and Nanotechnology, Pusan National University, Busan 46241, Korea; sangheon.jn@gmail.com (S.J.); 2jeong.s.o@gmail.com (J.J.); 2 Research Center for S-T/F, Samsung Electro-Mechanics, Busan 46754, Korea; vita_ebella@naver.com; 3 Department of Materials Engineering, Korea Aerospace University, Goyang 10540, Korea; 4 Smart Drone Convergence, Korea Aerospace University, Goyang 10540, Korea

**Keywords:** graphene, electrospinning, nanowire, etch-mask, nanoribbons, transistors

## Abstract

Graphene nanoribbons are a greatly intriguing form of nanomaterials owing to their unique properties that overcome the limitations associated with a zero bandgap of two-dimensional graphene at room temperature. Thus, the fabrication of graphene nanoribbons has garnered much attention for building high-performance field-effect transistors. Consequently, various methodologies reported previously have brought significant progress in the development of highly ordered graphene nanoribbons. Nonetheless, easy control in spatial arrangement and alignment of graphene nanoribbons on a large scale is still limited. In this study, we explored a facile, yet effective method for the fabrication of graphene nanoribbons by employing orientationally controlled electrospun polymeric nanowire etch-mask. We started with a thermal chemical vapor deposition process to prepare graphene monolayer, which was conveniently transferred onto a receiving substrate for electrospun polymer nanowires. The polymeric nanowires act as a robust etching barrier underlying graphene sheets to harvest arrays of the graphene nanoribbons. On varying the parametric control in the process, the size, morphology, and width of electrospun polymer nanowires were easily manipulated. Upon O_2_ plasma etching, highly aligned arrays of graphene nanoribbons were produced, and the sacrificial polymeric nanowires were completely removed. The graphene nanoribbons were used to implement field-effect transistors in a bottom-gated configuration. Such approaches could realistically yield a relatively improved current on–off ratio of ~30 higher than those associated with the usual micro-ribbon strategy, with the clear potential to realize reproducible high-performance devices.

## 1. Introduction

Graphene [1,2,3,4] exhibits far superior charge mobility (>250,000 cm^2^ V^−1^ s^−1^) which has been exploited to boost the performance of futuristic semiconductor electronic devices for a wide range of applications such as field-effect transistors (FETs) [5,6,7,8], sensors [9,10], supercapacitors [11,12], and nonvolatile memory [13,14]. However, an unusual linear energy–momentum dispersion relation in graphene results in zero bandgap [15], which is responsible for its semi-metallic behavior, and therefore restricts its utility in semiconductor technologies due to poor on–off current ratio at room temperature. To open a bandgap in graphene, various fabrication methods have been proposed, including doping, hydrogenation, nanoribbon or meshes in nanoscale, and even nanorings [16,17,18,19,20]. In particular, the transformation from graphene films to quasi-one-dimensional geometry (e.g., graphene nanoribbons (GNRs)) has garnered much attention as a new emerging research field [21,22,23]. Indeed, the breakthrough for GNRs has already been theoretically and experimentally evaluated by pioneers after the first manipulations with the exfoliated and integrated graphene device. Comprehensive theoretical models for GNRs firmly classified on the main principle with typical edge structures of the GNRs such as armchair- and zigzag-edge configurations, as reported previously [24,25,26]. The zigzag-edge configuration in GNRs possesses localized edge states that can spin-polarized to find application in spintronic devices such as spin valves [27], whereas the armchair-edge structure has non-magnetic semiconducting features with relatively larger bandgaps that increases with decreasing ribbon-width [28]. Thus, based on the theoretical and experimental results, the extension of graphene films to GNRs with high aspect ratios promises a value-added form of graphene by opening bandgap in quantum confinement of charge carriers, leading to the semiconducting nanomaterial. Nevertheless, a bandgap opening is still challenging because the width and the edge structure of GNRs should be controlled separately to determine the electronic properties of the devices built with the GNRs [29]. The relationship revealed so far is that the bandgap of GNR is inversely proportional to the width in its configuration, and the narrower armchair-edge structured GNRs are expected to express substantial charge mobilities, which often be considered as promising materials for electronic and optoelectronic applications [30]. Ideally, GNR-based FETs can be predicted with an improved electrical bandgap up to a few hundred meV, and relatively large *I*_On_/*I*_Off_ ratio at room temperature by narrowing the ribbon-width to few nanometer scales [31].

In this context, much effort has made on the fabrication process to evaluate the charge confinement and the edge effect on the electrical properties of the GNR-based FETs. The most commonly used method involved lithography to pattern the graphene film in nanoscale with a standard electron-beam (e-beam) source to produce individual or aligned arrays of GNRs. However, the e-beam exposure on the graphene surface often causes irradiation-induced damages and degrades the electronic transport properties of GNRs. It is therefore essential and timely to study efficient graphene patterning methods that define nanometer widths at high throughput without the use of e-beam irradiation or other cumbersome steps. To date, several advanced strategies have been suggested in patterning techniques using nanoscale masks such as metallic nanoribbons [32], nanowires [33], block copolymers [34], nanoimprinted polymers [35], self-assembled colloidal spheres [36], or even DNA molecules [37] to etch the exposed regions of graphene film with mild oxygen (O_2_) plasma. A simple route to produce GNRs is still required due to the complexity of multiple processing steps or other limitations in currently available manufacturing methods. Consequently, these technical issues inspire alternative nondestructive lithographical methods to assemble micro- or nano-objects into well-ordered structures, overcoming the fundamental and economic limitations.

Here, we developed a simple and efficient method to create aligned arrays of GNRs masked by highly oriented polymeric nanowires from the electrospinning process with a unique geometry. This utilization directed the polymeric nanowires in highly ordered configurations and allowed to fabricate robust etch-masks on a receiving substrate covered with the monolayer graphene produced by thermal chemical vapor deposition (CVD). The subsequent controlled O_2_ plasma process successfully matched the underlying graphene sheet on the nanowire etch-masks, facilitating the conformal secure protection from the reactive irradiation. Finally, the removal of polymeric nanowires with organic solvent constructed neat and clean edges of the GNRs on a substrate. On varying the polymer solution concentration in the electrospinning process, the size, morphology, and width of GNRs were easily manipulated on a large scale. Indeed, taking advantage of this approach, highly aligned arrays of GNRs could be built with micro-contact electrodes defined by conventional photolithography to integrate them into multi- or single-channel FETs. The *I*_On_/*I*_Off_ ratio for the FET fabricated from the extremely narrow GNR increased to ~30 on a single active channel, which suggest appreciable bandgap opening at room temperature. We believe that our simple strategy for the transfer printed nanopatterns of highly controllable polymeric nanostructures can extend their potential applications in cost-effective flexible electronics with easily accessible manner.

## 2. Materials and Methods

### 2.1. Polymer Solution Preparation and Electrospinning Process

The poly(methyl methacrylate) (PMMA, MW = 120 K, Sigma Aldrich, St. Louis, MO, USA) was dissolved in a mixed solvent of *N*,*N*-dimethylformamide (DMF, Sigma Aldrich, St. Louis, MO, USA) and acetone (6:4 ratio) to prepare the stock solution; the polymer solution was thoroughly stirred (800 rpm) at 40 °C for 12 h. For the electrospinning process, the stock solution was diluted in 10, 15, 17, 18, and 20 wt% by adding the additional solvent. A 10 mL polypropylene syringe with a 0.2 mm nozzle (inner diameter) was filled with the prepared PMMA solution, which was loaded into the electrospinning machine (ESR100, NanoNC, Seoul, Korea) with a constant feeding of the polymer solution (0.2 mL h^−1^) using a syringe pump. High voltage (15 kV, temperature: ~21 °C, relative humidity: ~45%) was then applied between the nozzle and the counterelectrodes (i.e., Cu bridged collector) at the optimum distance of 6.5 cm.

### 2.2. Synthesis of Graphene and Transfer on the Processing Substrate

The thermal CVD process was used to synthesize a graphene sheet from Cu foil (25 μm thick, 99.8%, Alfa Aesar, Ward Hill, MA, USA) using a typical tube furnace. As a first step, the annealing was performed for 50 min at 1050 °C under constant pressure (100 mTorr), and the mixture of H_2_/Ar (20/50 sccm) gas was flown for the reduction of Cu. Next, a reaction gas mixture (CH_4_/H_2_/Ar = 20/60/50 sccm) was introduced in the reaction tube for 20 min to grow the graphene sheet on the catalytic surface (i.e., Cu foil). Finally, the reaction tube was rapidly cooled (3 °C s^−1^) until it reached room temperature. For the graphene transfer on the desired substrate, PMMA solution (toluene, MW = 230 K, 10 wt%) was spun on the surface of a graphene/Cu foil (3000 rpm, 30 s). The PMMA coated graphene/Cu foil was baked on the hot plate at 70 °C for 10 min, and the Cu foil was then completely removed by using an etching solution (Transene Co., Inc., Danvers, MA, USA). After the removal of Cu foil, the PMMA/graphene film floating on the DI water was scooped and transferred onto the SiO_2_ (280 nm, thermal oxide)/Si substrates. The processing substrate was then placed on a hot plate at 130 °C for 15 min to evaporate the trapped water at the interface. Finally, the transfer medium film (PMMA) was removed by dipping in an organic solvent and rinsed with IPA.

### 2.3. Fabrication Process of FETs Using GNRs

For the integration of GNRs into FETs, standard photolithography (AZ-GXR 601, 14 cP, AZ Electronic Materials, Japan) was used. The arrays of the electric pads (i.e., source and drain) were defined on a SiO_2_ (dielectric)/highly doped Si (gate) substrate via thermally deposited Ti (5 nm) and Au (20 nm) and subsequent lift-off process. In the FET geometry, channel length (*L*_c_) and channel width (*W*) were designed with 3 μm and 80 μm, respectively. Separately, the CVD grown graphene sheet was transferred onto the pairs of the electrodes to form a tight conformal contact. Subsequently, arrays of the PMMA etch-mask (i.e., p-NWs) aligned on the graphene were then formed by using an electrospinning process. Finally, the O_2_ plasma etching process was performed on the prepared samples to remove the unprotected regions for 30 s at 100 W with O_2_ gas of 100 sccm to create GNR active channels between the electric pads. After the etching process, the p-NWs were fully removed by an organic solvent.

### 2.4. Characterization

The structured surface of the samples was observed by scanning electron microscope (SEM, Carl Zeiss AG-SUPRA 40 VP, 5–10 Kv, Germany) and optical microscope (Olympus BX51, Tokyo, Japan). The topological images were collected by using atomic force microscopy (AFM) in non-contact mode (XE-100, Park systems Corp, Suwon, Korea). The quality of graphene was verified by Raman spectroscopy with a laser excitation and spatial mapping process (UniRam-II, 532 nm, UniNanoTech, Seoul, Korea). The graphene-based FET measurement was carried out using a semiconductor parameter analyzer (Agilent 4156A, Santa Clara, CA, USA) in ambient condition. The transmittance of the graphene sheet was measured by UV-Vis (Evolution 201, Thermo Fisher Scientific Inc. Waltham, MA, USA).

## 3. Results and Discussion

**Figure 1:**
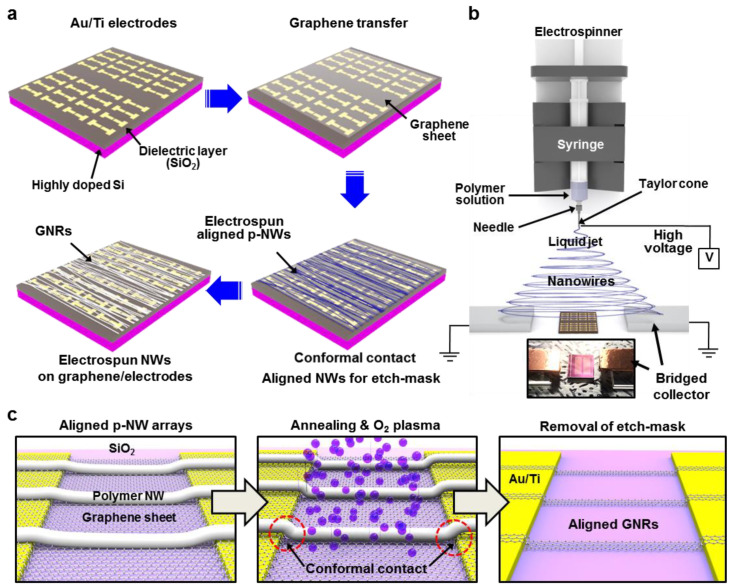
Schematic diagram of electrospinning-based graphene nanoribbons (GNR) fabrication and integration into a field-effect transistor (FET). (**a**) The electric pad preparation, graphene transfer, etch-mask formation, and the arrays of the GNR-based FETs (clockwise). (**b**) A schematic of the electrospinning set up to transfer the highly aligned arrays of p-NWs with the help of a bridged collector; the inset shows a real photograph of the process. (**c**) Annealing process for the conformal contact of the p-NW underlying the graphene sheet/electric pads to enhance the step-coverage, followed by O_2_ plasma etching and the removal of the p-NW with an organic solvent.

Figure 1a schematically illustrates the fabrication process to produce the aligned arrays of GNRs on the CVD grown graphene sheet, transferred on the electric contact pads, utilizing an electrospinning process in two-parallel bridged collector geometry [38]. The first step involved photolithography to define thermally evaporated source/drain electrodes (Au/Ti) on the highly doped Si wafer with SiO_2_ (280 nm) dielectric. Next, a CVD-grown monolayer graphene sheet was transferred onto the prepared electrode/SiO_2_/Si substrate, assisted by the thermal release tape or typical spin-casted polymer film [39]. After the extensive cleaning process with acetone and IPA, the electrospinning process was performed directly on the transferred graphene surface in the parallelly positioned bridged collector (i.e., Cu foil) to guide the electrospun nanowires (p-NWs) with high controllability as described in Figure 1b. This unique configuration of the bridged collector is simple, but innovative for the arrangement of the printed p-NWs on the prepared flat substrate that was placed between the nanowire collecting region. It should be noted that the key parameters in the process to create aligned arrays of p-NWs were to optimize with the distance between the nozzle and the receiving substrate confined in the two-parallel collectors that engaged in the horizontal shear flow onto the jetted polymer solution; the vertical distance between the nozzle tip (d = 33 μm) and the receiving substrate was precisely set at 6.5 cm with a relatively fast feeding of PMMA solution at constant speed (0.2 mL h^−1^) at the supplied voltage of 15 kV. The substrate covered with aligned arrays of p-NWs was then placed in the O_2_ plasma chamber to etch the unmasked area, followed by the removal of p-NWs using an organic solvent (i.e., toluene). Finally, the monolithically integrated GNR FETs in multichannel arrays could be produced with an electrically isolated configuration within the channel. As presented in Figure 1c, we hypothesized that the p-NWs could be formed slightly relief structure (left panel) presumably due to the gap between the electric pads (~25 nm thick) and the graphene surface. In fact, the monolayer of graphene sheet could be conformally transferred on the lithographically patterned electrode arrays without the critical undulation because of the ultrathin nature of thickness (i.e., ~0.36 nm) [40]. On the other hand, the stable entanglements to form the PMMA-NWs (i.e., ≥2.5 entanglements per chain), during the electrospinning of the polymer solution, influenced tight intermolecular interactions in the optimized viscosity (i.e., concentration) [41], which may produce non-contact regions at the sharp contact-edges of the prepared electric pads, as marked with dotted circles at both edges of the electrodes in the left panel in Figure 1c. This problem can be resolved with a simple annealing process above *T*_g_ of PMMA (140–150 °C) to be completely sagged by filling the surrounding nanogaps, prior to use the O_2_ plasma. This step enables to unfold randomly coiled or aligned chains of p-NWs (i.e., disentanglement) with the significantly increased mobility of the polymer chains [42]. The eventual structural relaxation of the polymer chains may induce partially slip at the surface and in the energetically favorable wetting environments (middle panel, Figure 1c). Finally, the stripping of the p-NWs using organic solvent yields the monolithic integration of multichannel GNR arrays firmly connected to the source/drain electrodes, as a form of bottom-gated FET configuration.

**Figure 2:**
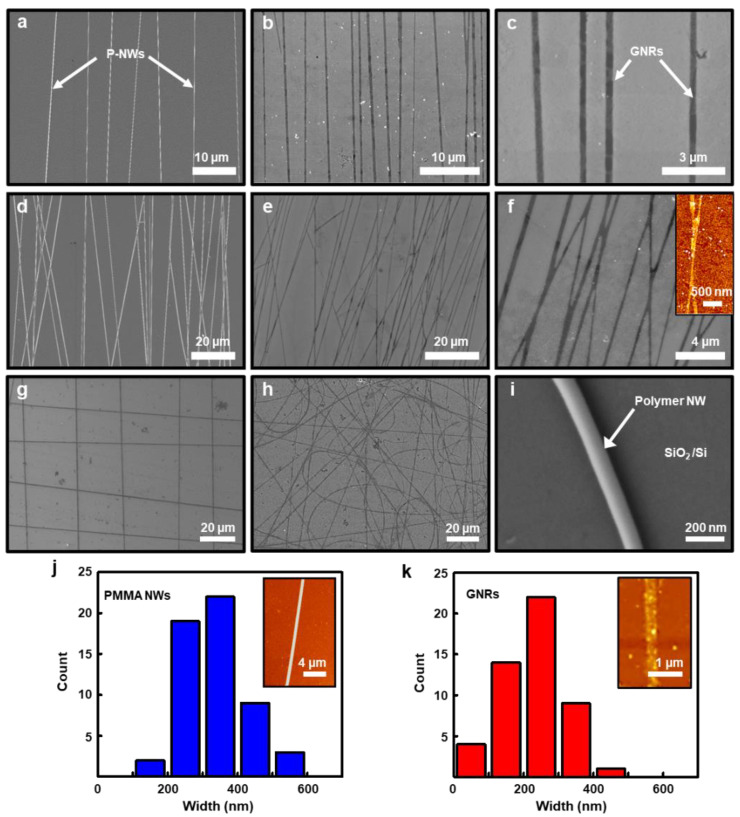
(**a**) SEM image of the aligned array of the electrospun p-NWs on a graphene sheet. (**b**,**c**) SEM images of the aligned array of GNRs after O_2_ plasma and the removal of p-NWs. (**d**) SEM image of the aligned-network arrays of the electrospun p-NWs on a graphene sheet. (**e**,**f**) SEM images of the GNR-networks; the inset shows an AFM image of the Y-shaped GNR. (**g**) SEM image of the orthogonal crossed GNRs. (**h**) SEM image of the random networks of GNRs. (**i**) the electrospun p-NW on SiO_2_/Si substrate. (**j**,**k**) Histograms of measured widths from the p-NWs (i.e., etch-mask) and processed GNRs, respectively; the insets show representative AFM height images.

Figure 2 presents a set of scanning electron microscope (SEM) images as a result of the control experiments for the optimization to fabricate the pattern-transfer of CVD-grown graphene sheets utilizing the p-NW based etch-masks. As appeared, the sequential process indicates that an array of GNRs was produced on the SiO_2_/Si substrate, where the perfectly matched features macroscopically observed with the underlying etch-masks after the O_2_ plasma process. To determine the width of the p-NWs was critical at the initial stage of the electrospinning experiment, especially for the highly aligned features, the typical trial-and-error methodology was employed to produce the p-NWs in bridged collector geometry. For this optimization, the concentration of polymer solution played an important role in controlling the alignment, width, and morphology of p-NWs onto a constringent collecting area. As guided by the previous reports, starting with a 10 wt.% concentrated PMMA solution, the nanowires were randomly distributed and disconnected in a short-wire form in the structure, while the regular nanowires appeared with a large number of bead-like morphologies were produced with the increased concentration (15 wt.%), and the appearance of such beads lasted until the concentration reached to 17 wt.%. With a slight modulation of the concentration of the polymer solution up to 18 wt.%, the beads disappeared, and eventually finely tuned requisite p-NWs could be generated in the range of 18 to 20 wt% (Figure S1). Figure 2a shows the aligned arrays of p-NWs formed on the graphene/SiO_2_/Si substrate with the initial condition (20 wt%). Subsequent O_2_ plasma (100 W, 30 s, 100 sccm) and removal of p-NWs with toluene successfully patterned the graphene sheet into the GNRs in a fast processing time (i.e., 6–7 min) as presented in Figure 2b,c. The corresponding GNRs were slightly misaligned with X-shaped interconnected formation in the ultrathin planar structures as a result of overlapped junctions of p-NWs etch-masks with less controllability, which was originated from the interaction of electrostatic forces between the long-ranged electrospun p-NWs. However, this unfavorable interference effect between the p-NWs could be simply resolved with the control of the electrospinning time (i.e., 5 min) that resulted in less nanowire deposition in the process on the same collector geometry. Therefore, as shown in Figure 2d–f, the p-NW-based lithography physically separated the individual GNR array with a highly aligned configuration, providing a potentially interference-free condition for use in the active channels of FETs. The local density of the GNR array was found to be ~3 per 10 μm with a slight local variation in the macroscopic view (Figure 2e), and the magnified SEM image clearly represents the discretely patterned GNR arrays (Figure 2f). In this experimental condition, the controllability further demonstrated by the second transfer of the highly aligned p-NWs on the same 90° rotated graphene substrate that produced a grid-pattern formation as appeared in Figure 2g and Figure S2. In addition, the random networks of graphene nanostructures were also fabricated from the flat collector without the use of the bridged geometry as shown in Figure 2h. The interconnected networks of the patterned graphene nanostructures in the right-angle configuration or random networks could be useful in transparent electrode or other possible applications [43]. The typical width of the single p-NW was identified by the magnified SEM image as shown in Figure 2i, in which the representative diameter was measured to be 201 ± 12 nm with smooth and neat edges. Moreover, by the measurement using atomic force microscope (AFM) on the selected area, histograms of the size of the p-NW etch-mask and the corresponding GNRs after the removal of the p-NW were statistically analyzed as shown in Figure 2j,k. Interestingly, the changes in the pattern-transition of GNR formation due to the O_2_ plasma etching effect were clearly evaluated, where the size distribution of the generated GNR was reduced by more than 30% over the entire arrangement, compared to the originally formed p-NW; the insets of the representative AFM images shows an examples.

**Figure 3:**
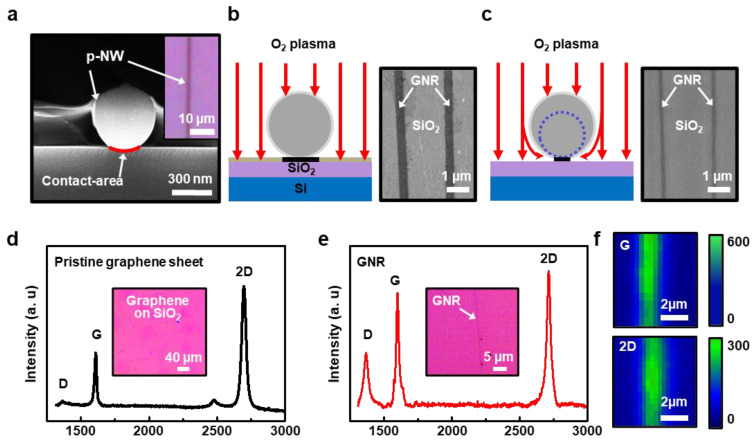
(**a**) SEM image of the side view of a p-NW; contact area was marked in red, and the inset shows an optical micrograph of a single p-NW on a graphene sheet. (**b**,**c**) O_2_ plasma process to remove the unprotected graphene sheet to engrave GNR arrays with different processing conditions. (**d**) Typical Raman spectrum of the graphene sheet on a SiO_2_/Si substrate, which indicates a monolayer in the graphene structure. (**e**) Raman spectrum measured from the GNR on a SiO_2_/Si substrate after O_2_ plasma and the removal of p-NWs. (**f**) Representative Raman mapping images of a patterned GNR (*w* = ~500 nm).

To explore the plasma etching effect for the optimized GNR fabrication, the correlation of the etch-masks (i.e., p-NWs) and the resulted width of GNRs were monitored as illustrated in Figure 3, which suggests a main parametric feature in our experimental scheme. Since the corresponding width of the GNRs is an important characteristic for the high-performance FET, it was postulated that the narrower GNR-width may be tailored by tuning the O_2_ plasma etching condition based on the estimated etch-resistance of the p-NWs. In fact, the electrospun nanowires were formed with cylindrical geometries on a flat surface in their nature; therefore, the effective masking regions should be defined only by the contact-area on the graphene surface. The cross-sectional view of the SEM image in Figure 3a obviously revealed the initial contact-area as marked in the red circle, implying the possible shadow effect in the O_2_ plasma environment. As reported previously, the profile of the sacrificial polymeric masking layer (e.g., photoresist) can be diminished, but utilized on the underlying patternable materials, maintaining the critical dimensions, by the accurate changes of the plasma settings [44]. In our case, the receiving graphene substrate masked by p-NWs can be etched more effectively as a function of the increased dissolution rate by controlling the processing parameter. Figure 3b,c demonstrates a reflected etching behavior on the morphological transformation, depending on the O_2_ plasma condition started with the identical width of the p-NWs on the graphene surface. Surprisingly, the much deeper plasma treatment (i.e., 100 W, 30 s, 100 sccm) reduced the width-scale of the GNRs from the original profile of 303 ± 23 nm to up to 120 ± 16 nm. It is important to note that once a perfect etch-mask array (i.e., p-NWs) was formed on the graphene surface with conformal passivation, the exposed graphene regions were firstly removed by O_2_ plasma, then the passivated p-NWs and graphene were simultaneously etched progressively via a rigorous ion bombardment (see the arrows in Figure 3c). As the etching rate of the p-NWs with a specific polymer molecular weight condition could be simply predicted by the approximation based on the previous reports [45], the plasma processing time was set depending on the initial diameter of p-NWs (i.e., width); otherwise, graphene can be completely etched or separated locally by the O_2_ plasma without forming a ribbon structure. As experimentally revealed, the SEM images in Figure 3b,c represent the changes of the GNRs width through the careful tuning of the O_2_ plasma process facilitating the p-NW-based etch-masks. Conclusively, to realize our scheme in defining the critical width of GNRs, the O_2_ plasma with a restricted processing time was a critical parameter as well as the control of the aligning of p-NWs. Raman spectroscopy was used to identify the quality of the CVD grown graphene and GNRs after the sequential patterning process. As investigated previously, the number of layers of graphene can be estimated by the relative intensity ratio of 2D and G peaks (i.e., *I*_2D_/*I*_G_) [46,47]. Figure 3d displays the characteristic 2D (2683 cm^−1^), G (1588 cm^−1^), and D (1352 cm^−1^) peaks, where the *I*_2D_/*I*_G_ found to be 1.82, indicating a monolayer graphene of high quality with a minimum detection of D peak; the inset shows an optical micrograph of the graphene transferred on the SiO_2_/Si substrate. As presented in Figure 3e, the initial integrity of graphene was maintained when measuring the GNR with ~500 nm. However, the intensity of the 2D peak was apparently increased from inherent defects at the devastated edges as a result of the sequential patterning and etching process. Raman mapping of 2D and G peaks in Figure 3f also demonstrates the spatial arrangement of the protected GNR on the SiO_2_/Si substrate, implying the effective removal of graphene exposed area to the O_2_ plasma process.

**Figure 4:**
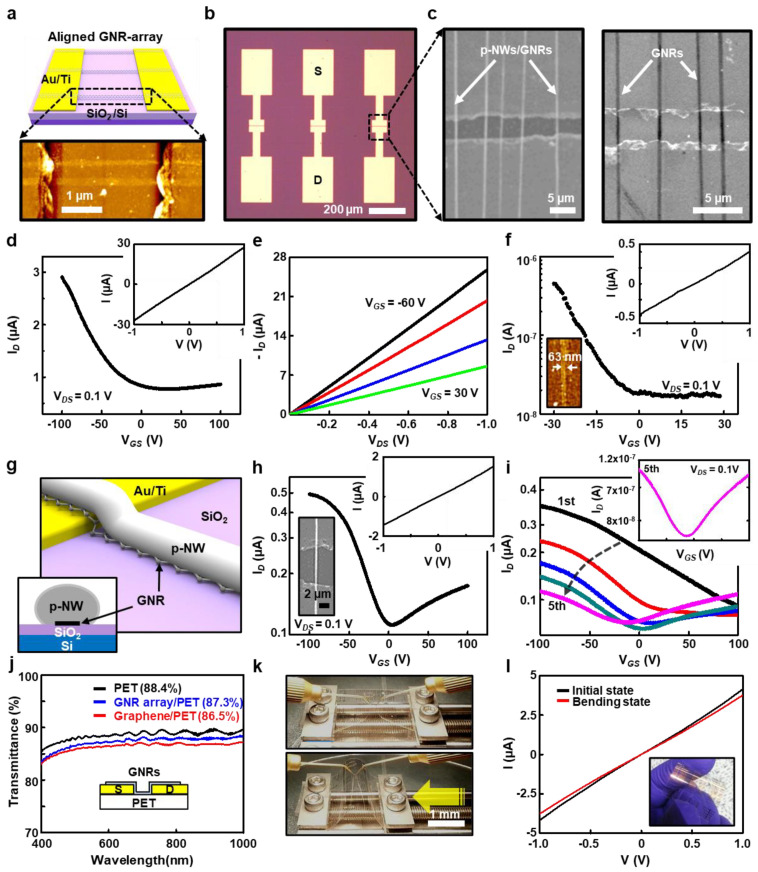
(**a**) Schematic of the bottom-gated GNR-FET (top) and the AFM images of the GNR array in between the electric pads (bottom). (**b**) Optical micrograph of the GNR-FET arrays. (**c**) The magnified SEM images of the p-NW etch-mask and GNRs in the active channel; all devices have ~4.7 μm channel lengths. (**d**,**e**) Drain current (*I*_D_) as a function of gate voltage (*V*_G_) for a multichannel GNRs (~20) in the source/drain bias (*V*_DS_) of 0.1 V, and the corresponding output characteristic of GNR-FET. (**f**) Representative transfer curve on a transistor that incorporates a single-channel of a GNR; the width was measured by AFM as ~63 nm (inset). (**g**) Schematic of the single-channel GNR, encapsulated by a p-NW (inset: cross-sectional view of GNRs in its longitudinal direction). (**h**) Typical transfer curve with charge neutral point centered at the charge-neutral point (inset: a representative SEM image of the single-channel GNR). (**i**) Ambipolar behavior of GNR-FET incorporated with the electric field annealing process. (**j**) The transmittance of the GNR-based two-terminal device on a PET substrate (inset shows a side view of the device). (**k**) Photographs for the demonstration of a flexible device placed in the bending stage. (**l**) Current density–voltage (*I*–*V*) characteristics for a representative device; linear fits define the effective resistance (inset: a photograph of the flexible device).

The patterned GNRs were readily integrated for bottom-gated FETs on a highly doped Si substrate as schematically illustrated in Figure 4a and Figure S3. The AFM image clearly confirmed aligned arrays of GNRs conformally structured over the contact pads of Au (20 nm)/Ti (5 nm). In Figure 4b, the optical micrograph shows electrically accessible pairs of the source/drain electrodes. The magnified SEM images of GNRs (before/after the removal of p-NWs) in between the channel length of 4.8 μm demonstrates the effective capability of the polymeric etch-mask on the rigorous O_2_ plasma, which leads to the electrically confined GNR-array with the width over the range from 80 to 230 nm (Figure 4c and Figure S4). Electrical transport studies on the bottom-gated device were carried out at room temperature under an ambient condition. Figure 4d presents a transfer characteristic of the GNR arrays FET with 3 lines per 10 μm (Figure S5). The device shows intrinsic *p*-type behavior with charge neutral point around 0 V. Figure 4e shows the output characteristics of the GNR array FET for various gate voltages, showing a typical linear region without stable saturation region like conventional GNR and graphene FET. In comparison, randomly oriented GNR array FET was also demonstrated in Figure S6. These GNR array FETs exhibited *p*-type behavior suppressing the electron conduction, which was presumably attributed to the Au electrode that lower the Fermi energy level in the GNR. The *p*-type behavior with Au contact was in good agreement with the previous report; the graphene became *p*-doped when contacted with the specific metals such as Au or Pt [48]. In addition, the edge oxidation or physisorbed O_2_ in the experimental conditions associated with the device fabrication process (e.g., O_2_ plasma and PMMA residues) may induce *p*-doped behavior in the graphene-based FETs. Though GNR array FET is preferred in terms of on-current characteristics, misalignment and slight variations in the width of the individual GNRs degrade quantum confinement of charge carriers by providing the multiple electron transport pathways.

Therefore, we have focused on the fabrication of single-channel devices through a short-period process (i.e., electrospinning a few seconds). When the O_2_ plasma flushed on the prepared substrate to reduce the initial diameter of the p-NWs at the same time as the graphene width, the extremely harsh conditions on the etch-mask enabled to create a single GNR channel formed between source/drain electrodes. Notably, the FETs consisting of 20 GNRs exhibited current on/off ratio of ~4 (Figure 4d), which is lower than that of ~30 in the single GNR FET as shown in Figure 4f; the AFM measurement revealed the GNR width of 63 nm (inset). The improved gate modulation of the single GNR FET can be explained by the fact that, as previously reported, narrowing the width of the graphene sheet can open the graphene bandgap with lateral charge carrier confinement (Table S1 (Supplementary Materials)). Nevertheless, the “on-state” current of the GNR-array FETs was higher than that of single GNR FET, which was one of the advantages of the multi-channel GNR FETs. In addition, the linear-regime mobility, *μ*, on the single-channel device can be calculated from the transfer curve, using the extracted peak transconductance [49]. As a typical model, we define the effective field-effect mobility of the FET device as

(1) $$\mu =\;∆\sigma /\left(C_{ox}∆V_{G}\right)\;=\left[\;\left(\frac{∆I_{D}}{V_{DS}}\right)\left(\frac{L}{W}\right)\right]/\left(C_{ox}∆V_{G}\right)$$

where *L* and *W* are the channel length and width, respectively; *C*_ox_ is the relative dielectric constant of the gate dielectric; and *I*_D_, *V*_DS_, and *V*_G_ are the drain-source currents, the drain-source voltage, and the gate voltage, respectively [49]. Within the device-to-device variations in properties, the typical hole mobility ranged from 80 to 120 cm^2^ V^–1^ s^–1^ for the single GNR channel devices. While the p-NWs used as a etch mask were removed for both single and GNR-array FET in Figure 4d–f, a single-channel GNR FET encapsulated with a p-NW was also presented for comparison in Figure 4g–i; in this process, the GNR FET sample was annealed in the vacuum oven at above *T*_g_ for 2 h (Figure 4g). Unlike the p-channel-dominant behavior of both single and GNR-array FETs, the n-channel also appeared from the single GNR FET covered with a p-NW etch-mask (Figure 4h). It was presumed that the Fermi energy level in the GNR move to a higher level through the perfect packaging in the polymer film that prohibited possible surface transfer doping by the oxygen adsorption on the GNR. In other words, the Fermi energy level of the GNR moved to close to the charge-neutral point of the GNR in the single GNR FET with the p-NW. Similar analysis for the multiple sweep with sufficiently high voltage yields ambipolar behavior as presented in Figure 4i and Figure S7. As previously reported [50], during this continuous electric field annealing, the physisorbed oxygen and contaminants on the GNR were removed by the electrothermal self-heating, so the n-channel mode was appeared and the charge-neutral point moved close to zero (i.e., the transfer curve in the inset). A more profound analysis of the array GNR FET was not performed at this work because the effect of precise edge roughness and geometry on the array GNR FET was unknown. To extend the available range of the application, we used a flexible plastic sheet (i.e., PET) as a substrate to demonstrate a two-terminal device with the electrode pairs (Figure 4j–l). When the transmittance was measured for each process, pseudo-transparent devices could be built on the PET sheet, representing the transparency over 87% in the optimized fill factor of metal electrodes (Figure 4j). The mechanical deformation on the device was tested using a bending stage to monitor the changes of the electrical properties (Figure 4k); no noticeable degradation of the electrical pathways was detected in the device structure (Figure 4l). Conclusively, we fabricated the aligned GNR array FET using a facial etch-mask made of controlled p-NW, which shows a decent transistor performance like that from conventional lithography approach. This approach will expedite the development of the GNR FET by providing statistical analysis of array GNR FET without an expensive and cumbersome process.

## 4. Conclusions

In summary, we developed a facile but robust method to fabricate GNRs by employing highly aligned arrays of polymeric etch-mask. Tailored configuration of p-NWs such as size, alignment, and width was fully demonstrated using a modified electrospinning process with a bridged collector. As a result, well-aligned narrow GNR has been produced by the p-NW protected O_2_ plasma etching process. Detailed characterization of the GNRs by AFM, SEM, and electrical measurements on single- and multi-channel devices reveal the key features of the related properties of the GNR arrays. The improved gate-modulation has proved a beneficial effect on devices built with the GNR arrays. Our strategy is compatible with various optimized dry etching processes described in the recent literature. Moreover, the p-NW could act as passivation masks underlying GNR to drive current capability and charge neutral point shift. The presence of both electron and hole conduction is a unique feature of our strategy to build GNR-based FETs. The current study represents the great potential of 2D electronics from graphene sheet to large-scale integration of GNRs into nanoelectronics [51].

## Data Availability

Data is contained within the article or supplementary material.

## Supplementary Materials

The following are available online at https://www.mdpi.com/2079-4991/11/1/33/s1. Figure S1: (a,b) Schematic illustration of the first and second alignment of p-NWs on a SiO_2_/Si substrate using a bridged collector. (c–e) Optical micrographs on the control experiments single, crossed, and random networks of p-NWs on the substrates. (f) Representative AFM image and corresponding height profile. (g) Concentration effect on the applied voltages in the electrospinning process, Figure S2: (a,b) SEM images of the random networks and orthogonally crossed GNR arrays on a SiO_2_/Si substrate after O_2_ plasma and the removal of p-NWs; the enlarged SEM images (right) represent randomly oriented and aligned GNR structures in the form of the interconnected configurations, Figure S3:(a) Schematic illustration of the electrospinning process on the processing substrate with electric pads (i.e., source/drain) to integrate GNR-array based FETs; a slide glass was used as a support in the transfer printing process of the highly aligned p-NWs. (b) The real images in the process of density control as a function of electrospinning time; the processing substrate was placed between the Cu electrodes (i.e., bridged collector), Figure S4: Annealing effect on the enhanced step coverage of GNRs in the active channel formation. (a) SEM image of the disconnected GNRs (see yellow arrows) between the source-drain electrodes without annealing process. (b) Conformally formed GNRs with electrically connected configuration between the source-drain electrodes with the annealing process. (c) AFM image measured from the sample in (a); GNRs were electrically isolated away from the electrodes. (d) AFM height profile measured from the sample in (b); the width of single GNR was ~82 nm in this case, Figure S5: Highly aligned arrays of GNRs between the source-drain electrodes; most of the GNRs was uniformly connected in the conductive channel, except some misaligned and disconnected on the electric pads. Figure S6: (a,b) Optical micrographs of the random network arrays of GNRs formed on a SiO2/Si substrate; the inset shows a representative AFM image measured from the surface, in which micron ribbons were appeared with unaligned configuration. (c,d) SEM images of partially aligned and unaligned arrays of GNRs between the source-drain electrodes; the yellow arrows indicate more widen GNRs, resulted from the overlapped etch-mask of the p-NWs. (e,f) Typical *I*_D_-*V*_G_ curve from the FET built with random networks of GNRs, and the corresponding output characteristics, Figure S7: Electric filed annealing effect on the GNR-FET. (a) *I*_D_-*V*_G_ curve of GNR device after high-power electrical annealing sweeps (i.e., 100 times), which shows slightly asymmetric hole and electron conduction. (b) *I*_D_-*V*_DS_ curve of the same device with 30 V steps of *V*_GS_.
