# Supplementary material for: Highly Aligned Polymeric Nanowire Etch-Mask Lithography Enabling the Integration of Graphene Nanoribbon Transistors
Source: Nanomaterials (Basel). 2020 Dec 25;11(1):33. doi: 10.3390/nano11010033 (PMC7824453; doi:10.3390/nano11010033)
Supplement: Supplementary file 1 [file nanomaterials-11-00033-s001.pdf]

Supporting Information

Highly Aligned Polymeric Nanowire Etch-Mask Lithography enabling the integration of Graphene Nanoribbon Transistors

Sangheon Jeon[1,†], Pyunghwa Han[2,†], Jeonghwa Jeong[1], Wan Sik Hwang[3,4*] and Suck Won Hong[1,*]

[1] Department of Cogno-Mechatronics Engineering, Department of Optics and Mechatronics Engineering, College of Nanoscience and Nanotechnology, Pusan National University, Busan 46241, Republic of Korea; sangheon.jn@gmail.com (S.J.); 2jeong.s.o@gmail.com (J.J.)

[2] Research Center for S-T/F, Samsung Electro-Mechanics, Busan 46754, Republic of Korea; vita_ebella@naver.com (P.H.)

[3] Department of Materials Engineering, Korea Aerospace University, Goyang 10540, Republic of Korea

[4] Smart Drone Convergence, Korea Aerospace University, Goyang, 10540, Republic of Korea

*Correspondence: whwang@kau.ac.kr (W.S.H.), swhong@pusan.ac.kr (S.W.H.)

†These authors contributed equally to this work.

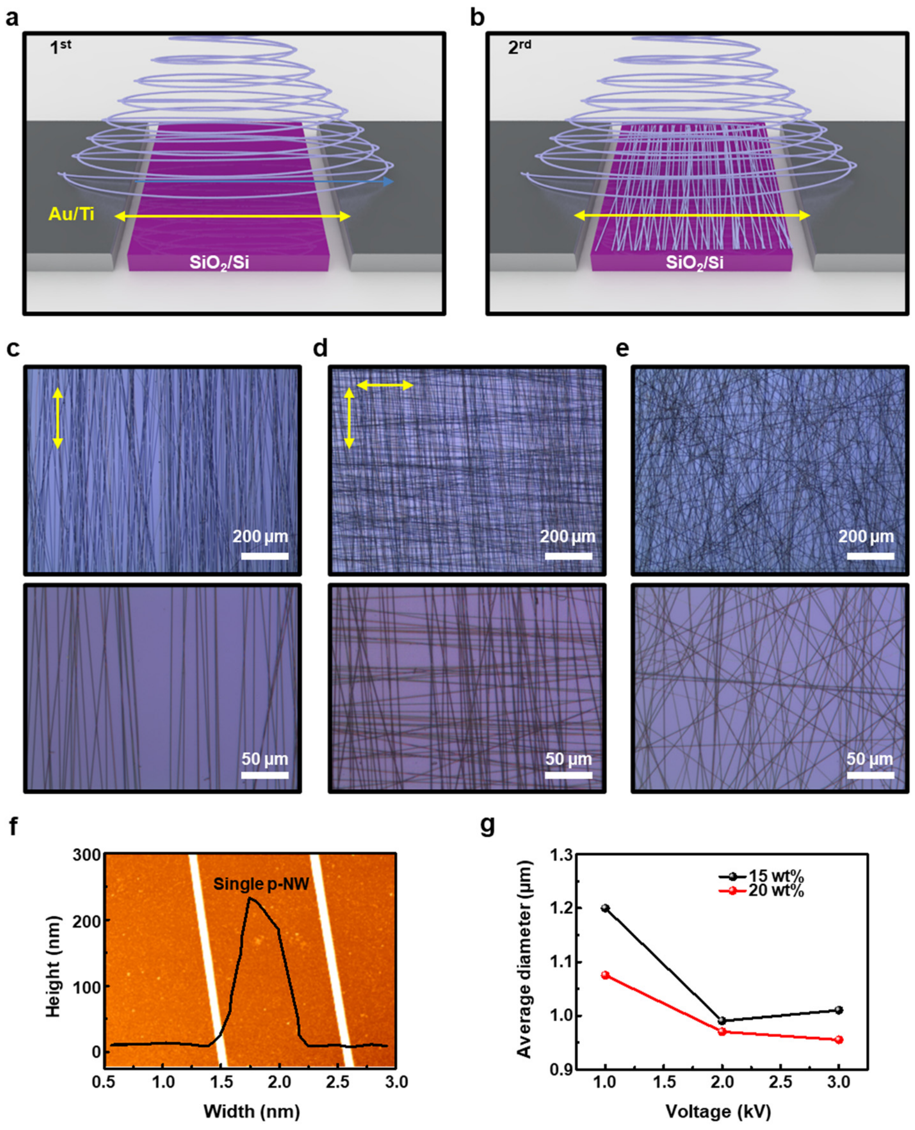

Figure S1. a-b) Schematic illustration of the first and second alignment of p-NWs on a SiO_2/Si substrate using a bridged collector. c-e) Optical micrographs on the control experiments single, crossed, and random networks of p-NWs on the substrates. f) Representative AFM image and corresponding height profile. g) Concentration effect on the applied voltages in the electrospinning process.

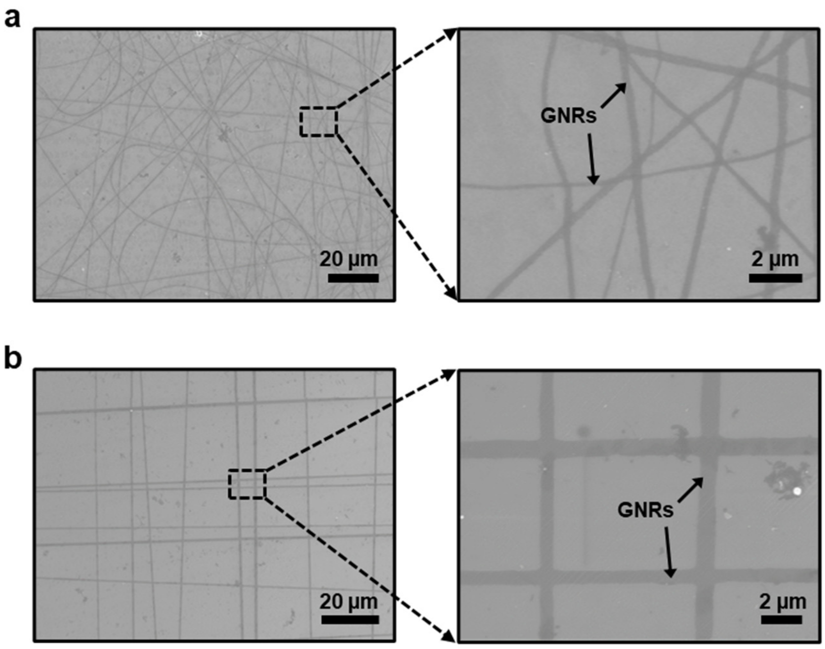

Figure S2. a-b) SEM images of the random networks and orthogonally crossed GNR arrays on a SiO_2/Si substrate after O_2 plasma and the removal of p-NWs; the enlarged SEM images (right) represent randomly oriented and aligned GNR structures in the form of the interconnected configurations.

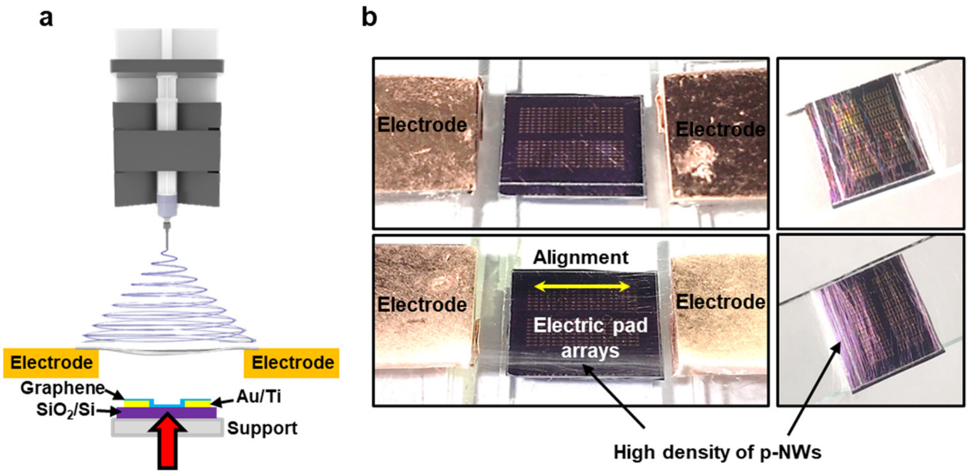

Figure S3. a) Schematic illustration of the electrospinning process on the processing substrate with electric pads (i.e., source/drain) to integrate GNR-array based FETs; a slide glass was used as a support in the transfer printing process of the highly aligned p-NWs. b) The real images in the process of density control as a function of electrospinning time; the processing substrate was placed between the Cu electrodes (i.e., bridged collector).

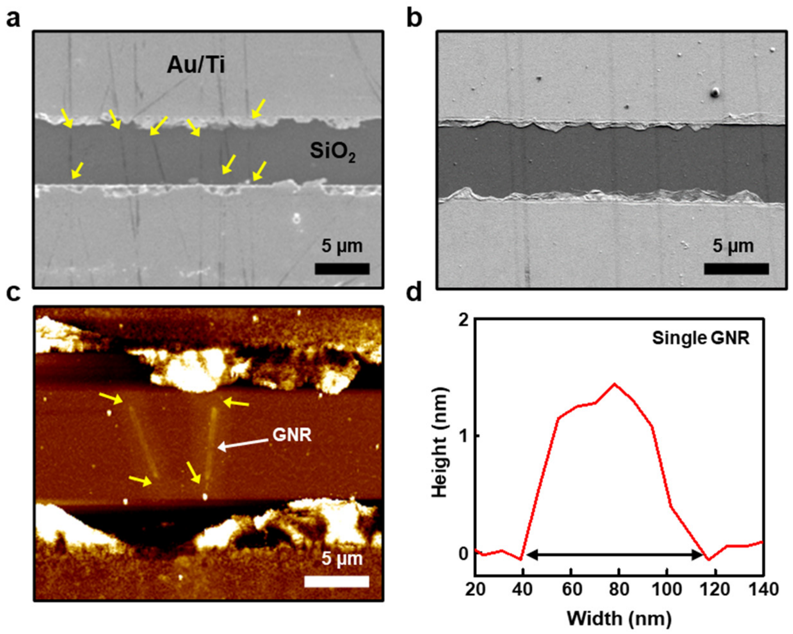

Figure S4. Annealing effect on the enhanced step coverage of GNRs in the active channel formation. a) SEM image of the disconnected GNRs (see yellow arrows) between the source-drain electrodes without annealing process. b) Conformally formed GNRs with electrically connected configuration between the source-drain electrodes with the annealing process. c) AFM image measured from the sample in a); GNRs were electrically isolated away from the electrodes. d) AFM height profile measured from the sample in b); the width of single GNR was ~82 nm in this case.

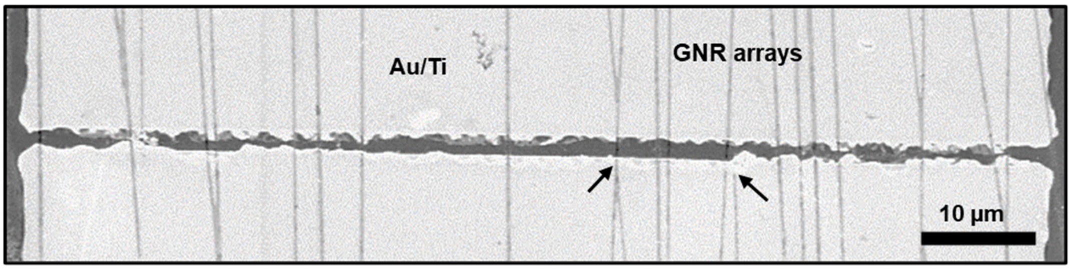

Figure S5. Highly aligned arrays of GNRs between the source-drain electrodes; most of the GNRs was uniformly connected in the conductive channel, except some misaligned and disconnected on the electric pads (see marked arrows).

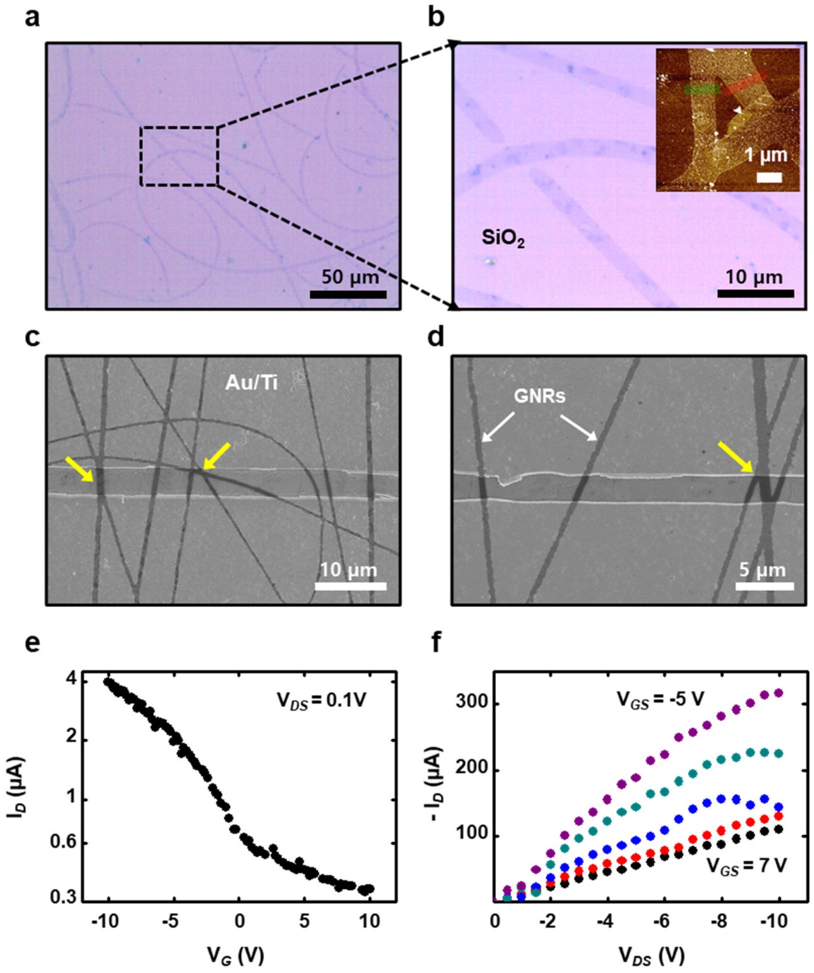

Figure S6. a-b) Optical micrographs of the random network arrays of GNRs formed on a SiO2/Si substrate; the inset shows a representative AFM image measured from the surface, in which micron ribbons were appeared with unaligned configuration. c-d) SEM images of partially aligned and unaligned arrays of GNRs between the source-drain electrodes; the yellow arrows indicate more widen GNRs, resulted from the overlapped etch-mask of the p-NWs. e-f) Typical I_D-V_G curve from the FET built with random networks of GNRs, and the corresponding output characteristics, respectively.

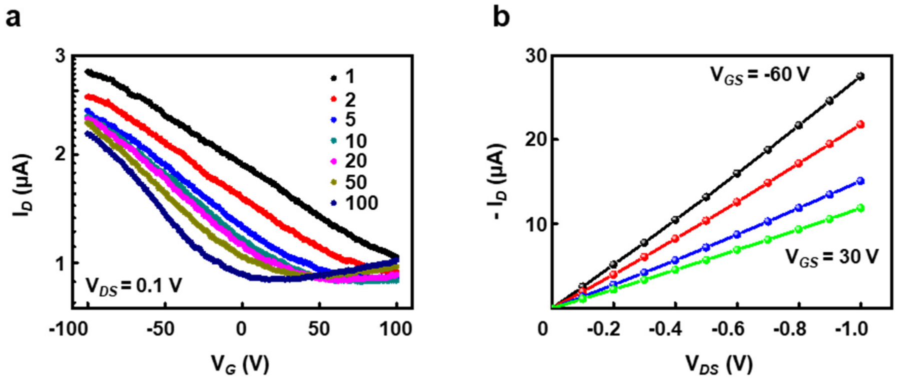

Figure S7. Electric filed annealing effect on the GNR-FET. a) I_D-V_G curve of GNR device after high-power electrical annealing sweeps (i.e., 100 times), which shows slightly asymmetric hole and electron conduction. b) I_D-V_{DS} curve of the same device with 30 V steps of V_{GS}.

Table S1. Previously reported GNR-based transistors.

Graphene width	I_{ON}/I_{OFF}	Method	Ref.
10 nm	$>10^5$	Chemical synthesis	1
10 nm	3000	E-beam lithography	2
15 nm	12	Nanowire lithography	3
~5 nm	270	Nanowire lithography	4
~40 nm	56	DNA nanowire array lithography	5
~10 nm	10	E-beam lithography	6

1. Li, X.; Wang, X.; Zhang, L.; Lee, S.; Dai, H. Chemically derived, ultrasmooth graphene nanoribbon semiconductors. *Science* **2008**, *319*, 1229-1232.
2. Yu, W. J.; Duan, X.; Tunable transport gap in narrow bilayer graphene nanoribbons. *Sci. Rep.* **2013**, *3*, 1248.
3. Liao, L.; Bai, J.; Lin, Y.-C.; Qu, Y.; Huang, Y.; Duan, X.; High-performance top-gated graphene-nanoribbon transistors using zirconium oxide nanowires as high-dielectric-constant gate dielectrics. *Adv. Mater.* **2010**, *22*, 1941-1945.
4. Liu,C.; Yao, B. ; Dong, T.; Ma, H.; Zhang S.; Wang, J.; Highly stretchable graphene nanoribbon springs by programmable nanowire lithography. *npj 2D Mater. Appl.* **2019**, *3*, 23.
5. Kang, S.H.; Hwang, W.S.; Lin, Z.; Kwon, S.H.; Hong, S.W. A Robust Highly Aligned DNA Nanowire Array Enabled Lithography for Graphene Nanoribbons Transistors. *Nano Lett.* **2015**, *15*, 7913-7920.
6. Hwang, W.S.; Zhao, P.; Kim, S.G.; Yan, R.; Klimeck, G.; Seabaugh, A.; Fullerton-shirey, S.K.; Xing, H.G.; Jena, D. Room-Temperature Graphene-Nanoribbon Tunneling Field-Effect Transistors. *npj 2D Mater. Appl.* **2019**, 1-7.
